# MicroRNA-138 Increases Chemo-Sensitivity of Glioblastoma through Downregulation of Survivin

**DOI:** 10.3390/biomedicines9070780

**Published:** 2021-07-06

**Authors:** Ji-Young Yoo, Margaret Yeh, Yin-Ying Wang, Christina Oh, Zhong-Ming Zhao, Balveen Kaur, Tae-Jin Lee

**Affiliations:** 1 Department of Neurosurgery, McGovern Medical School, The University of Texas Health Science Center at Houston, Houston, TX 77030, USA; Ji.Young.Yoo@uth.tmc.edu (J.-Y.Y.); Margaret.Yeh@uth.tmc.edu (M.Y.); Balveen.Kaur@uth.tmc.edu (B.K.); 2 Center for Precision Health, School of Biomedical Informatics, The University of Texas Health Science Center at Houston, Houston, TX 77030, USA; yingxiao8958@gmail.com (Y.-Y.W.); Zhongming.Zhao@uth.tmc.edu (Z.-M.Z.); 3 Department of Biosciences, Rice University, Houston, TX 77005, USA; chrissooh@gmail.com; 4 Human Genetics Center, School of Public Health, The University of Texas Health Science Center at Houston, Houston, TX 77030, USA

**Keywords:** glioblastoma (GBM), microRNA-138 (miR-138), BIRC5, survivin, temozolomide (TMZ)

## Abstract

Glioblastoma (GBM) is one of the most deadly cancers and poorly responses to chemotherapies, such as temozolomide (TMZ). Dysregulation of intrinsic signaling pathways in cancer cells are often resulted by dysregulated tumor suppressive microRNAs (miRNAs). Previously, we found miR-138 as one of tumor suppressive miRNAs that were significantly down-regulated in GBM. In this study, we demonstrated that ectopic over-expression of miR-138 sensitizes GBM cells to the treatment of TMZ and increased apoptotic cell death. Mechanistically, miR-138 directly repressed the expression of Survivin, an anti-apoptotic protein, to enhance caspase-induced apoptosis upon TMZ treatment. Using an intracranial GBM xenograft mice model, we also showed that combination of miR-138 with TMZ increases survival rates of the mice compared to the control mice treated with TMZ alone. This study provides strong preclinical evidence of the therapeutic benefit from restoration of miR-138 to sensitize the GBM tumor to conventional chemotherapy.

## 1. Introduction

Glioblastoma (GBM) is one of the most aggressive and lethal form of brain tumors [1]. Current treatment options, such as surgical resection, radiation, chemotherapy with temozolomide (TMZ), tumor-treating fields (TTFields) or their combination, only results in around 20 months of a median overall survival (OS) in patients [2]. Recurrence of GBM is nearly universal, and salvage therapies to impede further progression are ineffective [3]. Co-extinction strategies using multiple small molecule or antibody-based agents, however, are often hampered by drug-drug interactions, systemic toxicity due to pronounced off-target effects, and drug resistance. TMZ, a DNA alkylating agent, has become the standard chemodrug for GBM treatment [4]. However, therapeutic benefit from TMZ treatment is often modest in GBM patients. Rather, it only extends the long-term survival rates by a few months due to low concentration of TMZ accumulated in tumor cells and suppressed apoptosis. Major side effects of TMZ includes lymphopenia and non-specific toxicity [4]. Although TMZ is the FDA-approved chemotherapeutic option for primary GBM patients, the therapeutic benefit of TMZ needs to be further improved due to the poor response in GBM patients in current clinical settings. To overcome these limitations of TMZ for the treatment of GBM patients, tumor cells need to be sensitized to TMZ through reprogramming TMZ-related gene expressions.

MicroRNAs (miRNAs) have been implicated in many types of human cancers for their distinct role during the development, progression and metastasis [5]. Restoring tumor suppressive miRNAs in cancer cells has been proposed as one of potential therapeutic strategies as they can efficiently reprogram the aberrantly dysregulated cancer gene networks [6]. In the previous miRNA expression profiles obtained from small RNA sequencing on human GBM patient samples, we identified miR-138 as one of the most significantly down-regulated miRNAs in GBM [7]. In many studies, inhibition of Survivin, expressed from a gene *BIRC5*, expression was shown to enhance chemo-sensitivity of GBM to TMZ [8,9,10,11]. In addition, it was suggested that miR-138 directly down-regulates Survivin in certain types of cancers, such as bladder cancer, thoracic cancer and lung adenocarcinoma [12,13,14]. Therefore, we tested the hypothesis that miR-138 can increase the sensitivity of GBM cells to TMZ through the suppression of Survivin.

We observed that miR-138 over-expression sensitizes GBM cells to TMZ treatment with increased apoptotic cell death. Ectopic restoration of miR-138 negatively modulated the expression of Survivin in GBM cells leading to the increased survival rate in orthotopic GBM xenograft mouse model. We also observed that miR-138 directly regulates Survivin expression by binding to the 3′ UTR of *BIRC5* mRNA. Our data strongly suggest that the inhibition of Survivin through the restoration of miR-138 can benefit therapeutic outcomes from GBM patients in clinical settings.

## 2. Materials and Methods

### 2.1. Cells and Cell Cultures

Patient-derived de-identified primary GBM cells (GBM12, GBM28, and GBM43) were kindly provided by Dr. Jann N. Sarkaria (Mayo Clinic, Rochester, MN, USA), and their use was approved by The University of Texas Health Science Center at Houston (UTHealth, Houston) Institutional Review Board (IRB). The patient-derived primary GBM cells were maintained as tumor spheres in neurobasal medium supplemented with 2% B27 without vitamin A, human EGF (20 ng/mL), and basic FGF (20 ng/mL) in low-attachment cell culture flasks. All cell cultures were maintained at 37 °C in a humidified atmosphere with 5% carbon dioxide (CO_2_). All cells were routinely monitored for morphology changes and mycoplasma contamination, and authenticated by Short Tandem Repeat (STR) analysis at the University of Arizona Genetics Core.

### 2.2. Cell Proliferation Assay

Patient-derived primary GBM cells were plated in 6-well plates at density of 5 × 10^5^ cells per well in DMEM/F12 containing 2% FBS, 2% B27 without vitamin A, human EGF (20 ng/mL), and basic FGF (20 ng/mL) without antibiotics. The cells were transiently transfected with miR-138 mimics (Dharmacon miRIDIAN microRNA Mimics, Horizon Discovery, USA) or negative control mimics after pre-incubated with Lipofectamine RNAiMAX transfection reagent (Invitrogen, Thermo Fisher Scientific, USA) by following the manufacturer’s manual. Then the cells were incubated at 37 °C in a humidified atmosphere with 5% carbon dioxide (CO_2_). Four days later, each well was treated with equal volume of CellTiter-Glo Luminescent Cell Viability Assay. After 10 min of incubation, the plate was examined by Synergy H1 multi-mode microplate reader (BioTek Instruments, USA) for the luminescence intensity, which is proportional to the number of live cells.

For live cell proliferation imaging, engineered GBM12 cells expressing red fluorescence protein (GBM12-RFP) was transiently transfected with miR-138 mimics as described above. The cells were then treated with TMZ (Sigma-Aldrich, St. Louis, MO, USA) at the final concentration of 10 µM for GBM12 and GBM43, while 50 µM for GBM28 (Supplementary Figure S1). Using Cytation-5 Live Cell Imaging System (BioTek Instruments, Winooski, VT, USA), the live cells were imaged every four hours by visualizing fluorescence intensity from the RFP-positive GBM12-RFP cells with excitation at 532 nm and emission at 588 nm.

### 2.3. Apoptosis Analysis

GBM cells were transfected with miR-138 mimics or miR-Ctrl as described above in 6 well plates in the presence of 10 µM TMZ or DMSO. After four days of transfection, the GBM cells were stained with FITC-Annexin V/PI Apoptosis Detection Kit (BD Biosciences, San Diego, CA, USA) according to the manufacturer’s manual, and subjected to flow cytometry by Beckman CytoFLEX Flow Cytometer (Beckman Coulter, Indianapolis, IN, USA). The obtained flow cytometry data was analyzed with FlowJo version 10 software (BD Biosciences, San Diego, CA, USA). Only Annexin V-positive cells were considered to be apoptotic cell populations.

### 2.4. Western Blotting

GBM cells harvested from in vitro or GBM tumor from in vivo studies were denatured in RIPA buffer (Pierce Biotechnology, Waltham, MA, USA). The cell lysates were fractionated by 4–20% Criterion TGX SDS-PAGE Pre-cast gels (Bio-Rad, Hercules, CA, USA), then transferred to 0.45 µm Amersham Protran nitrocellulose (NC) membranes (Sigma-Aldrich, St. Louis, MO, USA). After blocking with 5% BSA-containing TBS blocking buffer, the NC membranes were probed with primary antibodies diluted at 1:1000 against Survivin, p-H2AX, cleaved Caspase-3, cleaved PARP, or GAPDH (Cell Signaling Technology, Waltham, MA, USA) followed by further incubation with HRP-conjugated secondary antibodies (GE Healthcare, Piscataway, NJ, USA). The immuno-reactive bands were visualized using enhanced chemiluminescence (ECL) (GE Healthcare, Piscataway, NJ, USA) by Chemi-Doc gel imaging system (Bio-Rad, Hercules, CA, USA).

### 2.5. Dual Luciferase Reporter Assay

Firefly/Renilla Duo-Luciferase reporter vector expressing the 3′ UTR clone of human *BIRC5* containing a putative binding site of miR-138 was purchased from GeneCopoeia, USA. A mutation was introduced into the putative miR-138 binding site by replacing adenines and guanosines within the seed sequences to cytosines using site-directed mutagenesis kit (GenScript, New Jersey, NJ, USA). The plasmid DNAs were amplified from *E.coli* were, then purified by DNA Maxi kit (Qiagen, Germantown, MD, USA). GBM cells plated in 12 well plates one day before were transfected with 1 μg of *BIRC5* 3′ UTR reporter DNA, or *BIRC5*-Mut DNA in Lipofectamine 3000 (Invitrogen, Thermo Fisher, USA). Next day, the cells were further transfected with miR-138 or miR-Ctrl as described above. After 48 h post transfection, the cells were lysed with Passive Lysis Buffer (Promega, Madison, WI, USA) and assayed with Dual Luciferase Assay kit (Promega, Madison, WI, USA) according to the manufacturer’s instruction.

### 2.6. Animal Studies

Six to eight weeks old NSG mice (NOD-scid IL2Rgamma-null) (Jackson Laboratory, Bar Harbor, ME, USA) were housed and handled in accordance with the guideline of UTHealth Center for Laboratory Animal Medicine and Care (CLAMC) and the animal protocols approved by the UTHealth Animal Welfare Committee (AWC). Intracranial glioblastoma xenograft was generated in the NSG mice by implanting GBM cells as previously described [15,16].

For in vivo survival studies, GBM28 cells were stably transduced with human pre-miRNA Expression Lenti-miR Vector containing the full-length miR-138 and green fluorescent protein (GFP) gene (System Biosciences, Palo Alto, CA, USA). Human pre-miRNA Scramble Negative Control Expression Lenti Vector plasmid was used as a control. Pre-miR-138 and negative control vector plasmids were packaged with pPACKH1 Lentivector Packaging Kit (System Biosciences, Palo Alto, CA, USA) in HEK293TN packaging cells according to the manufacturer’s manual. The transduced GBM cells were sorted by FACS analysis (FACSCalibur, BD Biosciences, San Diego, CA, USA) to select GFP-positive cell population containing pre-miRNA. The expression of miR-138 in the sorted cells was further confirmed by quantitative real-time PCR with TaqMan miRNA Expression kit (Applied Biosystems, Thermo Fisher Scientific, Waltham, MA, USA). Total of 5 × 10^5^ GBM28 cells were implanted into seven NSG mice brain for each group. The mice were daily treated with 10 mg/kg of TMZ on days 10–14 post tumor implant via oral gavage.

For western analysis, tumor tissues from the mice brain were harvested after three weeks from the intracranial implantation, and snap frozen in liquid nitrogen. For histological analysis, the harvested tumor tissues were fixed in 4% PBS-saturated formaldehyde. The fixed tissues were embedded in paraffin and sectioned at 5 µm thickness. Representative sections from each group were stained with Survivin or Ki67 antibodies (Cell Signaling, USA) by immunohistochemistry (IHC).

### 2.7. Statistical Analysis

All statistics was analyzed with GraphPad Prism 7 software (GraphPad Software, San Diego, CA, USA). Data were analyzed using unpaired, two-tailed *t*-tests for the comparison of the difference between two groups. Kaplan–Meier survival curves were compared by using the log-rank test. Statistical significance was determined by *p*-value lower than 0.05. Data variations in a group were expressed as mean ± SD (standard deviation).

## 3. Results

### 3.1. miR-138 Sensitizes GBM to Chemotherapeutic Drug Temozolomide

**Figure 1:**
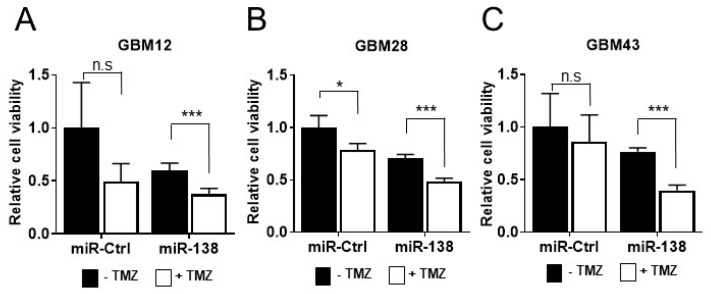
Combination of TMZ with miR-138 over-expression increases inhibition of cell proliferation in vitro. Patient-derived primary GBM cells, (**A**) GBM12, (**B**) GBM28 and (**C**) GBM43, were transfected with miR-138 mimics or negative control (miR-Ctrl). After 4 days of transfection, viable cells were measured by CellTiter-Glo Luminescent Cell Viability Assay. All error bars indicates standard deviations (*n* = 3), and the *p*-values were determined by two-tailed student *t*-test. * *p* < 0.05, *** *p* < 0.001, n.s = not significant.

**Figure 2:**
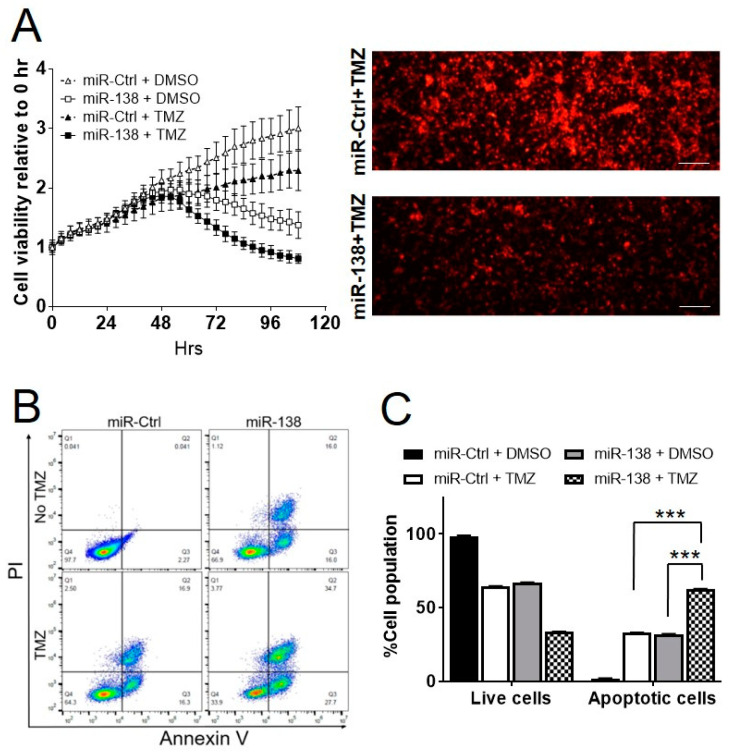
Over-expression of miR-138 combined with TMZ inhibits cell proliferation and increases apoptosis in vitro. (**A**) Cell proliferation analysis by fluorescence live cell imaging every four hours on GBM12-RFP cells after transfection of 25 nM miR-138 or miR-Ctrl. Left, the plot shows live cells imaged every 4 h to detect fluorescence intensity; Right, representative fluorescence images of GBM12-RFP cells at 96 h after the treatment of miRNA and TMZ. Scale bars indicate 100 µm. (**B**) Apoptosis analysis on GBM28 cells after transfection of 25 nM miR-138 or miR-Ctrl followed by Annexin V-PI double staining for flow cytometry. The scale bar indicates 100 µm. (**C**) Annexin V-positive cell population was considered to be apoptotic cells from cytograms. All error bars indicates standard deviations (*n* = 3), and the *p*-values were determined by two-tailed student *t*-test. *** *p* < 0.001.

Previously, we reported that ectopic expression of miR-138 in GBM patient-derived primary cells (GBM 12, 28 and 43) abolishes their cell growth, demonstrating the tumor suppressive potential of miR-138 in GBM [7]. To assess an impact of miR-138 overexpression on chemo-sensitivity in GBM cells, three GBM cells (GBM12, 28 and 43) were transiently transfected with 25 nM of miR-138 mimics in the presence or absence of TMZ (10 µM for GBM12 and GBM43; 10 µM for GBM28; Supplementary Figure S1) in comparison to scrambled negative control (miR-Ctrl). After 4 days from the transfection, all GBM cells transfected with miR-138 showed significant level of sensitivity to TMZ in comparison to of the cells treated with miR-138 alone (GBM12, *p* = 0.002; GBM28, *p* = 0.0001; and GBM43, *p* = 0.0002) (Figure 1). However, GBM cells treated with miR-Ctrl showed no (GBM12, *p* = 0.070; and GBM43, *p* = 0.521) or little sensitivity (GBM28, *p* = 0.017) to the treatment of TMZ (Figure 1). The results indicates that the combination of TMZ with miR-138 overexpression increases chemo-sensitivity of GBM cells. Using GBM12-RFP, engineered GBM12 cells to express red fluorescence protein, the cell growth was monitored by live cell imaging for over 4 days after treatment with 25 nM of miRNA and 10 µM of TMZ. Transient transfection of miR-138 mimics significantly inhibited the proliferation of GBM12-RFP cells after 48 h post transfection, in accordance to the previous report [7]. The combination of miR-138 overexpression with TMZ inhibited cell proliferation at more significant level than miR-138 alone (Figure 2A). In the same experiment, GBM12-RFP cells treated with miR-Ctrl with or without TMZ did not decrease the cell growth at significant level (Figure 2A). When the cells were subjected to flow cytometry analysis after stained with Annexin V-PI double staining, the GBM cells co-treated with 25 nM of miRNA and 10 µM of TMZ presented nearly twice apoptotic cells (62.6 ± 0.3%) compared to miR-138 (32.9 ± 0.3%) or TMZ alone (32.0 ± 0.3%) (Figure 2B,C, *p* < 0.001). All of these in vitro experimental data clearly demonstrated that combination of TMZ with miR-138 over-expression can increase the cytotoxicity of TMZ in GBM cells.

### 3.2. BICR5 Is Downregulated in Glioblastoma

**Figure 3:**
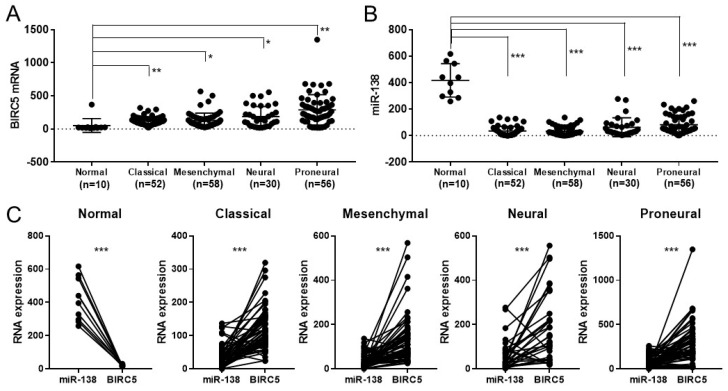
The Cancer Genome Atlas Program (TCGA) database shows inverse correlation between *BRIC5* and miR-138 in their expression. (**A**) *BIRC5* expression is up-regulated, while (**B**) miR-138 expression is down-regulated in most types of glioma compared to that of normal tissues. (**C**) Direct comparison between *BIRC5* and miR-138 clearly reveals inverse correlation in all types of glioma compared to normal tissues. All error bars indicates standard deviations, and Student *t*-test was used to determine the significance in difference between the two groups. * *p* < 0.05, ** *p* < 0.01, *** *p* < 0.001.

To unveil the molecular mechanism behind the synergistic combination between TMZ and miR-138 overexpression, TCGA database was mined for the correlation of expressions between miR-138 and *BIRC5*. Compared to the RNA expression in normal tissues (53.24 ± 104.6, *n* = 10), mRNA expression of *BIRC5* was up-regulated in most types of glioma patients: classical (120.7 ± 64.42, *n* = 52); mesenchymal (130.0 ± 110.6, *n* = 58); neural (186.7 ± 154.8, *n* = 30); and proneural (289.6 ± 228.4, *n* = 56) (Figure 3A). In contrast, the expression level of miR-138 was significantly lower in the most types of glioma patients: classical (35.32 ± 35.44, *n* = 52); mesenchymal (32.81 ± 32.53, *n* = 58); neural (63.04 ± 71.26, *n* = 30); and proneural (82.1 ± 68.13, *n* = 56), compared to normal tissues (417.3 ± 126.4, *n* = 10) (Figure 3B). When the expression levels of miR-138 and *BIRC5* were compared in each type of glioma, it was inversely correlated in all types of glioma at significant level (Figure 3C). These results indicate that miR-138 is down-regulated in glioma at an inverse correlation with *BIRC5*. In addition, the gene set enrichment analysis (GSEA) analysis on RNA-seq data obtained from human GBM patient samples (NCBI GEO database GSE165286 [7]) revealed that the genes participating in the pathways “negatively regulating DNA damage stimulus response” (NES: 1.85, FDR: 0.004) (Supplementary Figure S2) and “negatively regulating apoptosis” (NES: 1.90, FDR: 0.002) (Supplementary Figure S3) were highly expressed in GBM, implying the involvement of Survivin during the insensitivity of GBM to DNA damaging agent, such as TMZ.

### 3.3. Survivin Is a Direct Target of miR-138

**Figure 4:**
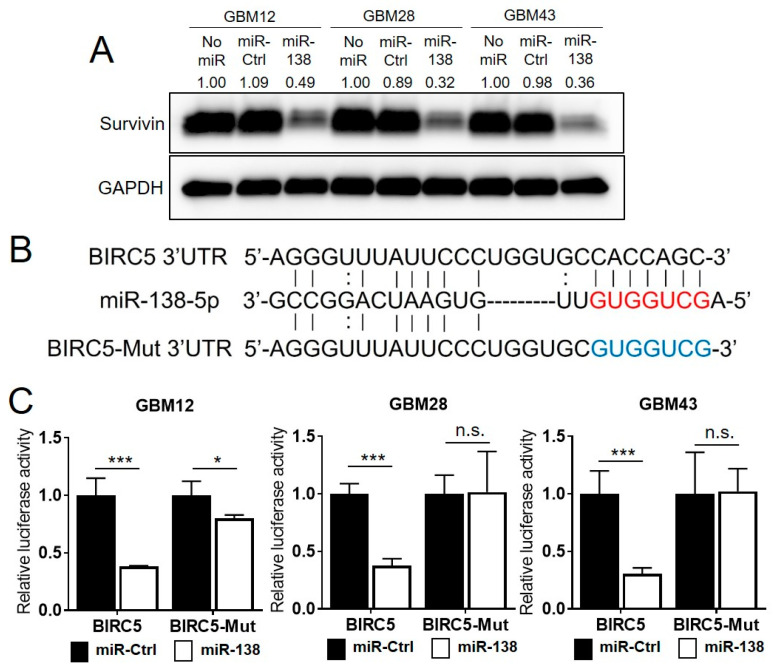
miR-138 negatively regulates the expression of Survivin through direct targeting its 3′ UTR. (**A**) Western blotting on GBM cells (GBM12, GBM28 and GBM43) with transient overexpression of miR-138. The protein expression levels of Survivin were significantly decreased by imR-138 compared to miR-Ctrl treated GBM cells. Relative fold change of Survivin expression by miR-138 or miR-Ctrl was compared to the No miR control in each cell. (**B**) Schematic diagram of 3′ UTR sequences of Survivin containing a predicted miR-138 binding site (red letters). BIRC5-Mut construct contains the mutated sequences in the seed regions (blue letters). (**C**) Luciferase reporter assays for direct binding of miR-138 to the 3′ UTR of BIRC5. GBM cells were co-transfected with miR-138 and the luciferase reporter plasmid DNA containing BIRC5 3′ UTR, or BIRC5-Mut sequences respectively. The cells were further treated with TMZ for 4 days, and the repression of luciferase activity by miR-138 was analyzed by Dual Luciferase Assay kit. The relative luciferase activity values were normalized to Renilla luciferase activity as internal control. All error bars indicates standard deviations (*n* = 3), and the *p*-values were determined by two-tailed student *t*-test. * *p* < 0.05, *** *p* < 0.001, n.s = not significant.

The inverse correlation between the expression of miR-138 and *BIRC5* suggests a possibility that miR-138 directly target *BIRC5*. In western blotting on GBM cell lysates, transient overexpression of miR-138 showed a significant reduction of the Survivin expression compared to GBM cells treated with miR-Ctrl (GBM12, 28 and 43) (Figure 4A). Target gene repression by miRNAs is mediated through a direct binding of miRNA to the 3′ UTR of target gene mRNA. Bioinformatics prediction software search (TargetScan, version 7.2 (Released March 2018): http://www.targetscan.org/vert_72/, accessed on 20 June 2020) revealed that the 3′ UTR region of human *BIRC5* contains a putative binding site matched with the seed sequence of miR-138-5p (Figure 4B) [13]. A luciferase vector plasmid was constructed to contain the 3′ UTR sequence of human *BIRC5* to carry out dual luciferase reporter assay. Additional luciferase reporter vector was also constructed to contain mutated seed sequences within the putative miR-138 binding sites (BIRC5-Mut) (Figure 4B). GBM cells (GBM12, 28 and 43 cells) were co-transfected with miR-138 mimics and the luciferase reporter vector plasmids. Luciferase activity was significantly decreased by miR-138 in all three GBM cells transfected with wild type BIRC5 3′ UTR (GBM12, *p* = 0.002; GBM28, *p* = 0.00003; and GBM43, *p* = 0.0005) (Figure 4C). However, the reduction of luciferase activity was not observed in the GBM cells transfected with BIRC5-Mut plasmids (GBM12, *p* = 0.02; GBM28, *p* = 0.94; and GBM43, *p* = 0.92) (Figure 4C), indicating that the direct interaction between miR-138 and the 3′ UTR region of *BIRC5* was responsible for the reduction of luciferase activity with sequence specific manner. Our data clearly showed that *BIRC5* is a direct target of miR-138, which down-regulates the expression of Survivin through direct binding to the 3′ UTR region of *BICR5*.

### 3.4. Ectopic Expression of miR-138 Improves Survival Rates in Intracranial Tumor Bearing Mice

**Figure 5:**
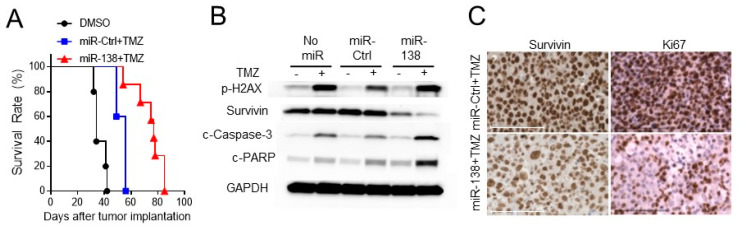
Sensitization of GBM cells by miR-138 reduces tumorigenicity in orthotopic in vivo model. (**A**) Mouse survival Kaplan-Meier survival curve for mouse survival rates. Intracranial xenograft tumor was induced by implanting GBM cells transduced with miR-138 or miR-Ctrl expressing lentiviruses. (**B**) Mice brain tumor tissues were harvested subjected to western blotting to show the expression levels of Survivin. Expression of cleaved Caspase-3 and cleaved PARP was detected to show activation of apoptosis. Phosphorylated H2AX (p-H2AX) was used as an indicator for the DNA damage activity of TMZ. (**C**) Representative immunohistochemistry staining images of mice brain tumor tissue sections for the change of Survivin expression by miR-138. Ki76 was stained for cell proliferation. Scale bars indicate 100 µm.

To evaluate the preclinical impact of miR-138 on the resistance of GBM tumors to TMZ, intracranial GBM tumor was induced in NSG mice by implanting GBM28 cells after transduced with either miR-138 or miR-Ctrl. GBM28 was chosen to test, since it was relatively more resistant to TMZ than GBM12 or GBM43 cells (Supplementary Figure S1). From day 10 to 14 post the tumor implantation, the mice were daily treated with TMZ. Kaplan–Meier survival curves showed that the combination treatment of TMZ with miR-138 significantly increased survival rates of the GBM-bearing mice (median survival of 77 days) compared to that of mice treated with TMZ and miR-Ctrl (median survival of 56 days, *p* = 0.0076) or DMSO alone (median survival of 34 days, *p* = 0.003) (Figure 5A). From western blotting analysis on the harvested mice GBM tumor, it was confirmed that the expression of Survivin was lowered by miR-138 overexpression (Figure 5B). Conversely, miR-138 overexpression elevated the expression levels of cleaved Caspase-3 and cleaved PARP as an indicative of apoptosis in the TMZ-treated mice brain (Figure 5B). However, miR-Ctrl did not significantly alter the TMZ-induced activation of those apoptosis effectors compared to no-miR controls (Figure 5B). IHC staining on the mice GBM tumor tissues also revealed the down-regulation of Survivin expression while increased expression of Ki67 was observed by miR-138 restoration (Figure 5C). All of these results clearly demonstrated that miR-138 restoration sensitizes GBM cells to TMZ by inducing apoptotic cell death through the direct repression of Survivin.

## 4. Discussion

This study aimed to identify a role of putative tumor suppressive miR-138 during chemo-resistance of GBM. As discussed earlier, we have found from recent RNA-seq on human GBM patient samples that miR-138 can play a tumor suppressive role when overexpressed in GBM cells and extend survival rates in GBM mice model systems [7]. The finding was in accordance with many previous studies as shown that an overexpression of miR-138 can suppress cell proliferation, metastatic ability and drug resistance of cancer cells in many types of cancers [17,18,19].

For many years, activation of Survivin has been implicated in human cancers including GBM and responsible for inactivation of Caspase pathway. Survivin is a pro-survival oncogene that is highly overexpressed in cancer cells and inhibits caspase activation to block an induction of apoptosis [20,21]. In addition, miR-138 was previously shown to directly target Survivin in bladder cancer and esophageal cancer [13,14]. We recognized an inverse correlation in the expression between miR-138 and *BIRC5* in all types of glioma. Our data also showed that miR-138 overexpression sensitizes GBM cells to TMZ through direct down-regulation of Survivin by binding to its 3′ UTR. Our study results clearly indicate a strong rationale of combination of miR-138 over-expression with TMZ treatment for therapeutic benefit in GBM patients. Interestingly, it was reported that miR-138 can educate CD4+ T cells by down-regulating two immune checkpoints, programmed cell death 1 (PD-1) and cytotoxic T-lymphocyte-associated molecule 4 (CTLA-4), which resulted in 43% increase of survival rates in GBM mice models [22]. It can be also expected to observe increased tumor mutation burden (TMB) through TMZ-mediated DNA damages in the sensitized tumor, which will result an increase of neoantigen formations to further activate immune response [23]. Therefore, it will be intriguing to combine the TMZ treatment with T cell activity by single tumor suppressor, miR-138, for synergistic anti-tumor effect. For future translation into human clinical trials, miR-138 needs to be efficiently delivered into GBM cells by distinguishing adjacent normal cells. Recently advanced RNA nanotechnology will be useful to test the challenging task [24,25]. The RNA-driven small RNA delivery system has shown the preclinical potential for successful targeted delivery of cargo RNAs into brain tumor cells across BBB via folate (FA)-mediated folate receptor (FR) recognition on tumor cells [15,16,26].

In contrast, a compelling result was reported that miR-138 may act as an oncogenic miRNA and increase TMZ resistance in glioma [27]. They demonstrated that an ectopic expression of miR-138 promoted TMZ resistance by targeting apoptosis regulator BIM. Their observation was obtained from long term-established glioma cell lines, which may display different characteristics from the patient-derived primary GBM cells that were used for this study. Therefore further study will need to clarify the role of miR-138 in TMZ resistance, and whose GBM patients will benefit from the restoration of miR-138.

## 5. Conclusions

We report that putative tumor suppressive miRNA, miR-138, can sensitize GBM tumor by negatively modulating Survivin. The results strongly demonstrated the therapeutic potential of miR-138 for primary GBM tumor by itself or through a combination with TMZ, which needs to be further assessed through future clinical trials.

## Data Availability

The data presented in this study are available on request from the corresponding author.

## Supplementary Materials

The following are available online at https://www.mdpi.com/article/10.3390/biomedicines9070780/s1.
