# Supplementary material for: MicroRNA-138 Increases Chemo-Sensitivity of Glioblastoma through Downregulation of Survivin
Source: Biomedicines. 2021 Jul 6;9(7):780. doi: 10.3390/biomedicines9070780 (PMC8301402; doi:10.3390/biomedicines9070780)
Supplement: Supplementary file 1 [file biomedicines-09-00780-s001.zip › biomedicines-1223251-supplementary.pdf]

biomedicines

MDPI

Supplementary Materials

MicroRNA-138 Increases Chemo-sensitivity of Glioblastoma through Downregulation of Survivin

Ji Young Yoo [1], Margaret Yeh [1], Yin-Ying Wang [2], Christina Oh [3], Zhong-Ming Zhao [2,4], Balveen Kaur [1] and Tae Jin Lee [1,*]

[1] Department of Neurosurgery, McGovern Medical School, The University of Texas Health Science Center at Houston, Houston, TX 77030
[2] Center for Precision Health, School of Biomedical Informatics, The University of Texas Health Science Center at Houston, Houston, TX 77030
[3] Department of Biosciences, Rice University, Houston, TX 77005
[4] Human Genetics Center, School of Public Health, The University of Texas Health Science Center at Houston, Houston, TX 77030
* Correspondence: Tae Jin Lee (Tae.Jin.Lee@uth.tmc.edu)

Keywords: Glioblastoma (GBM), microRNA-138 (miR-138), BIRC5, Survivin, temozolomide (TMZ)

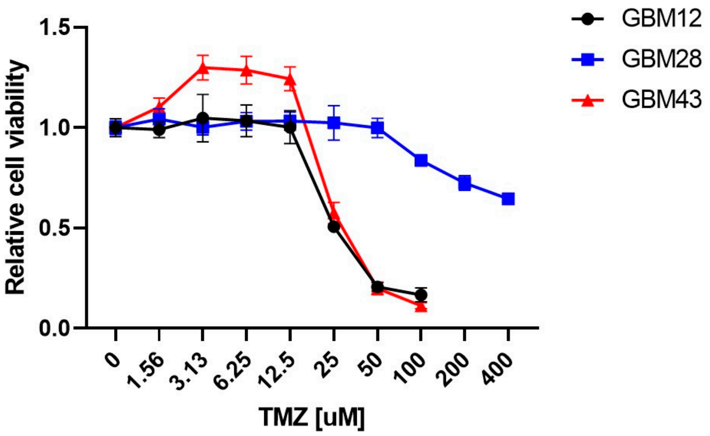

Supplementary Figure S1.

Cell viability assay to determine the sensitivity of GBM cells to the treatment of TMZ. GBM12, 28 and 43 cells were treated with a series of concentration of TMZ ranging from 0 to 400 μM in 24 well plates. After 96 hrs, cell viability was measured by CellTiter-Glo Luminescent Cell Viability Assay (Promega, USA). All error bars indicates standard deviations (n=4).

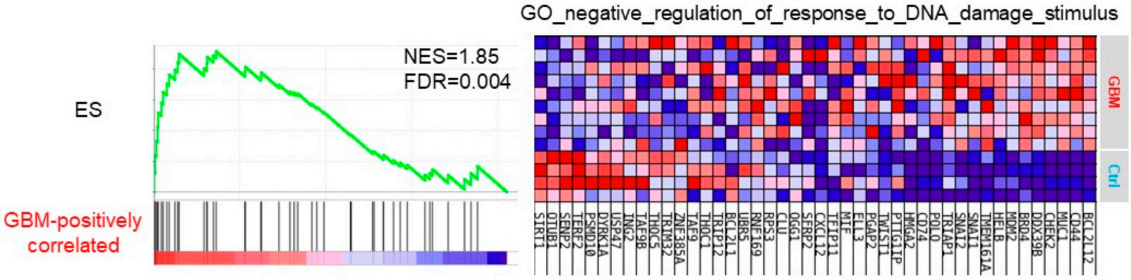

Supplementary Figure S2.

Enrichment plot by Gene Set Enrichment Analysis (GSEA) on the RNA-seq data obtained from human GBM patient samples (NCBI GEO data-base GSE165286) revealed showed the highly enriched scores (ES) in the genes participating in "negatively regulating DNA damage stimulus response". The normalized enrichment score (NES) was 1.85, and false discovery rate (FDR) was 0.004.

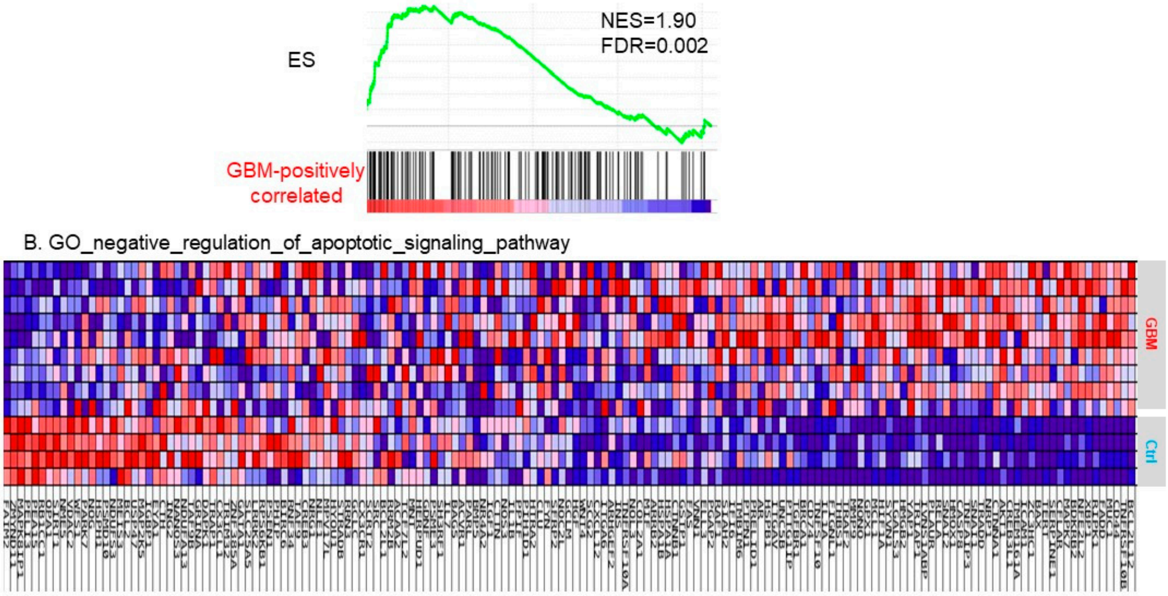

Supplementary Figure S3.

Enrichment plot by Gene Set Enrichment Analysis (GSEA) on the RNA-seq data obtained from human GBM patient samples (NCBI GEO data-base GSE165286) revealed showed the highly enriched scores (ES) in the genes participating in "negatively regulating apoptosis". The normalized enrichment score (NES) was 1.90, and false discovery rate (FDR) was 0.002.
